# Effect of prenatal lifestyle intervention on maternal postpartum weight retention and child body mass index z-score at 36 months

**DOI:** 10.1038/s41366-021-00784-8

**Published:** 2021-02-24

**Authors:** Suzanne Phelan, Chantelle N. Hart, Elissa Jelalian, Karen Muñoz-Christian, Noemi Alarcon, Angelica McHugh, Alison K. Ventura, Rena R. Wing

**Affiliations:** 1 California Polytechnic State University, Department of Kinesiology & Public Health, Center for Health Research, San Luis Obispo, CA USA; 2 Temple University Center for Obesity Research and Education Department of Social and Behavioral Sciences, Philadelphia, PA USA; 3 Warren Alpert Medical School at Brown University Department of Psychiatry and Human Behavior, Providence, RI USA; 4 California Polytechnic State University World Languages & Cultures Department, San Luis Obispo, CA USA

**Keywords:** Weight management, Phase III trials

## Abstract

**Background/Objectives:**

We previously reported results from a randomized trial showing that a behavioral intervention during pregnancy reduced excess gestational weight gain but did not impact maternal weight at 12 months. We now examine the longer-term effects of this prenatal intervention on maternal postpartum weight retention and toddler body-mass-index z scores (BMIz) over 36 months.

**Subjects/Methods:**

Pregnant women (*N* = 264; 13.7 weeks’ gestation; 41.6% Hispanic) with overweight or obesity were randomized into usual care or prenatal intervention. Anthropometric assessments in mothers and toddlers occurred at baseline, 35 weeks’ gestation and after delivery at 6, 12, 18, 24, and 36 months.

**Results:**

At 36 months, prenatal intervention vs. usual care had no significant effect on the proportion of participants who returned to their early pregnancy weight or below (33.3% vs. 39.5%; *p* = 0.12) and had no effect on the magnitude of weight retained (2.8 [0.8, 4.8] vs 3.0 kg [1.0, 4.9], respectively; mean difference = 0.14 [−3.0, 2.7]). There was also no statistically significant intervention vs. usual care effect on infant BMIz or skinfold changes over time; toddler BMIz increased by 1.4 [−1.7, 1.0] units in the intervention group and 1.6 [−1.2, 1.8] units in the usual care group from delivery to 36 months (difference = 0.16 [−0.32. 0.63]). The proportion of toddlers at risk for obesity at 36 months was similar in intervention and usual care groups (28/77 [36.4%] vs 30/80 [37.5%]; *p* = 0.77).

**Conclusions:**

Compared with usual care, lifestyle intervention during pregnancy resulted in similar maternal and toddler anthropometric outcomes at 36-months postpartum in a diverse US sample of women with overweight and obesity. To sustain improved maternal weight management initiated during pregnancy, continued intervention during the postpartum years may be needed.

## Introduction

Excess weight gain during pregnancy is a well-documented predictor of high postpartum weight retention in women [1, 2] and child obesity during infancy, toddlerhood, and adolescence [3]. In women with obesity, lifestyle interventions during pregnancy can reduce excess gestational weight gain [4] and 12-month postpartum weight retention [5]. Interventions that continue during the postpartum year appear to have stronger effects on reducing 12-month postpartum weight retention [5]. However, it is unclear whether effective lifestyle interventions delivered during pregnancy have enduring impacts on maternal postpartum weight retention and child body mass index (BMI) beyond the first year, particularly in women with overweight/obesity [6].

Healthy Beginnings/Comienzos Saludables was a randomized clinical trial of lifestyle intervention with meal replacements to reduce excess gestational weight gain in Hispanic and non-Hispanic women with overweight and obesity [7]. The intervention was effective in reducing excess gestational weight gain in both Hispanic and non-Hispanic women [8]. The intervention compared with usual care resulted in lower mean weekly gestational weight gain (0.33 vs. 0.39 kg/wk; *p* = 0.02) and reduced the proportion of women who exceeded National Academy of Science (NAS) guidelines for total gestational weight gain (41% vs. 54%; *p* = 0.03). The intervention stopped after delivery and had no significant effect on 12-month postpartum weight retention [8] or infant BMIz [5]. Prior research has suggested that prenatal interventions might not affect child weight outcomes until later in life [9].

The purpose of this study was to test the longer-term effects of the Healthy Beginnings/Comienzos Saludables lifestyle intervention. The primary hypothesis was that women randomized to a prenatal lifestyle intervention versus usual care would have reduced weight through 36-months postpartum and their offspring would have lower weight status and adiposity (body mass index z scores, BMIz; skinfolds) through age 36 months and lower cumulative incidence of obesity.

## Materials/Subjects and methods

### Design

**Fig. 1:**
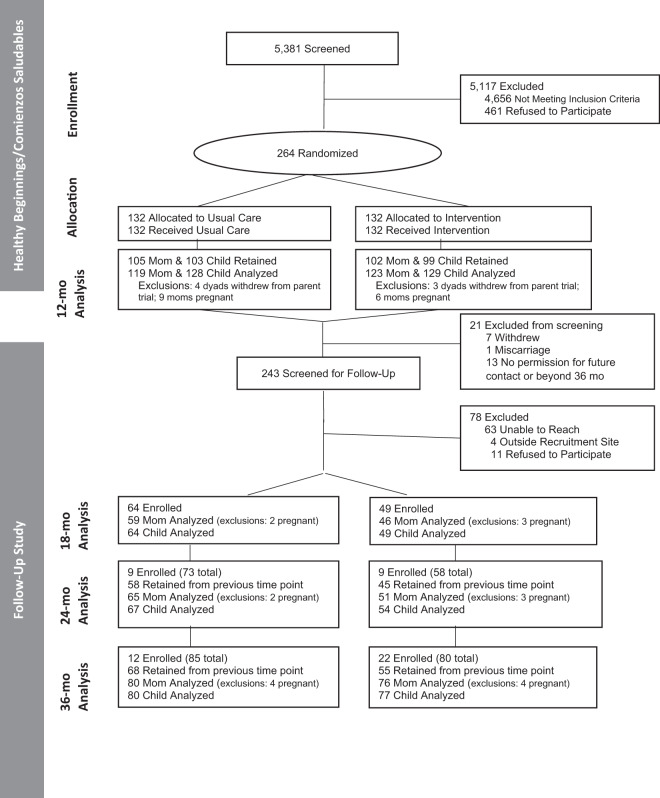
Participant flow into Healthy Beginnings/Comienzos Saludables and the follow-up study. Enrollment in the follow-up study was ongoing, and some participants did not enroll for postpartum follow-up until 24 or 36 months.

This follow-up study was secondary to the Healthy Beginnings/Comienzos Saludables randomized clinical trial. The trial was conducted at California Polytechnic State University, San Luis Obispo, California, and at the Miriam Hospital with Women & Infants Hospital in Providence, Rhode Island. It was part of the Lifestyle Interventions for Expectant Moms (LIFE-Moms) consortium [10]. Healthy Beginnings/Comienzos Saludables’ recruitment, eligibility, assessments [7], and effects during pregnancy [7] and at 12-months postpartum [8] have been reported previously. In the original trial, participants consented to follow-up through 12-months postpartum. Additional funding was obtained to continue to follow Healthy Beginnings/Comienzos Saludables participants and their children through 36 months with enrollment and assessments being completed at 18, 24, and 36-months post-delivery. Given the time lag between the end of Healthy Beginnings/Comienzos Saludables and commencement of this follow-up study, some mother/child dyads missed the opportunity to enroll at 18 or 24 months; however, in these cases, efforts were made to obtain chart-abstracted weight from physician offices (Fig. 1).

### Participants

Procedures were approved by Institutional Review Boards, and all participants provided written informed consent. Eligibility criteria included gestational age between 9 and 16 weeks, BMI ≥ 25, English or Spanish-speaking, age ≥18 years, and singleton pregnancy. Exclusion criteria included major physical or mental health problems, history of bariatric surgery, contraindications to aerobic exercise, loss of contact during initial screening, and other less frequent criteria. An additional eligibility criterion for the follow-up included child ≤36 months at study entry.

### Interventions

Randomization was computer generated by the study statistician, and women were randomly assigned within site (California vs. Rhode Island) and ethnicity (Hispanic vs. non-Hispanic) to one of the two treatment conditions: (1) usual care or (2) behavioral lifestyle intervention with partial meal replacement during pregnancy.

### Usual care

Women in usual care attended their regularly scheduled visits with their prenatal care providers [11]. Women also attended a brief visit at study entry and received newsletters at 2-month intervals through 12-months postpartum and postcards yearly for the remainder of the study to promote retention in the study.

### Behavioral lifestyle intervention with partial meal replacement during pregnancy

Participants in the intervention group received all aspects of usual care plus a behavioral lifestyle intervention with partial meal replacement during pregnancy. As described previously [7], the intervention encouraged women to gain approximately one-half pound (0.23 kg) per week [12]. Each woman received ~20 min, individual, face-to-face counseling sessions with a study interventionist every two weeks until 20 weeks gestation and then monthly until delivery. The interventionist discussed appropriate weight gain during pregnancy, physical activity (30 min of walking most days of the week), and behavioral strategies, including daily self-monitoring. At each prenatal intervention visit, a supply of meal replacement shakes and/or bars were provided free of charge, and women were instructed to replace two meals with meal replacement products per day in addition to at least one meal of regular foods and two to four healthy snacks each day [7]. Body weight scales, food records, and pedometers were also provided to promote adherence to daily self-monitoring. After delivery, the intervention was discontinued and meal replacements were no longer provided.

### Assessments

Demographic and weight history information were obtained at baseline. Race and ethnicity, demographic factors, and childbearing history were assessed by self-report using questionnaires with fixed categories. A stadiometer was used to measure maternal height in duplicate to the nearest 0.1 cm. At all assessment points (i.e., baseline, 35 weeks’ gestation and at 12, 18, 24, and 36-months postpartum), maternal weight was assessed by research assistants (masked to randomization) in duplicate to the nearest 0.1 kg using a calibrated standard digital scale with the participant in lightweight clothing without shoes. Women were categorized as exceeding or not exceeding the NAS recommendations for total gestational weight gain [12]. Net postpartum weight retention from baseline was defined as the difference between study measured maternal weight at baseline and weight measured at the 36-month postpartum visit. Net postpartum weight retention from pre-pregnancy weight was also computed and defined as the difference between maternal self-reported pre-pregnancy weight and study-measured weight at the 36-month postpartum visit. Validity of self-reported pre-pregnancy weight was supported by high correlations with weight measured early in pregnancy (*r* = 0.97; *p* = 0.0001), albeit slightly lower in women with lower (<50,000/year) vs. higher (≥50,000/year) household incomes (*r* = 0.95 vs *r* = 0.99) and in Hispanic vs. non-Hispanic women (*r* = 0.95 vs *r* = 0.98). Percent weight retention was defined as postpartum weight retention divided by the starting weight and multiplied by 100.

Child weight, length/height, and skinfold thicknesses were measured by trained research assistants (masked to randomization) at birth (within 14 days) and at 6, 12, 18, 24, and 36-months postpartum. Weight was measured using a calibrated scale and length (through 18 months) was measured using a standardized board; standing height was measured at 24 and 36 months with a portable stadiometer. All assessments were performed in duplicate and if the values differed by a specified amount (>0.1 kg for weight, >0.5 cm for length/height), a third measurement was taken. The average of the closest two measurements was used in data analyses. BMI z-scores were calculated using the WHO Child Growth Standards for age and sex [13]. A z score of >1 was used to define at risk for obesity. Skinfold thickness was measured by trained staff in duplicate using the Harpenden skinfold caliper on right side of the body at the following sites: triceps, subscapular, thigh and iliac crest. Participants received compensation for completing assessments that included measures for a feeding study (videotaping a mealtime interaction) as follows: $25 for study entry, 35 weeks’ gestation, and 6 months postpartum; $50 for 12-months postpartum; $75 for 18 and 24 months postpartum; and, $50 for an abbreviated 36-month postpartum visit.

### Statistics

*T*-tests and chi-square tests were used to compare women who completed the 36-month assessment visit with women who did not complete this visit and also to compare the intervention and usual care group participant characteristics. Likelihood-based, linear mixed effects models were used to assess the effects of treatment group on maternal weight, simultaneously adjusting for pre-specified potential effect modifiers that included weeks’ gestation at randomization, age, ethnicity (Hispanic vs. non-Hispanic), parity (multiparity vs. primiparity), study entry weight, household family income (≥50,000/year vs. <50,000/year), and site (California vs. Rhode Island). Model fit (using −2 Log Likelihood) did not improve in quadratic versus linear modeling; thus, linear modeling was retained in all analyses. Participants missing baseline covariate data (*N* = 3) were not included in analysis. Otherwise, all participants were included in the mixed effect models, and any missing data were handled using maximum likelihood estimation. For analysis of follow-up data, multiple linear regression models were used to examine the impact of the intervention on weight changes within specific time intervals (i.e., during pregnancy, between 6 and 12 months, etc.), adjusting for the same potential effect modifiers. A priori general linear models with covariates were examined to determine group differences in postpartum weight retention from baseline (i.e., net and percent postpartum weight retention), adjusting for the same potential effect modifiers. A multiple logistic regression analysis was used to examine the effect of treatment group on the proportion of women who achieved baseline weight or below at 36-months postpartum. Similar secondary analyses examined group effect on weight over time relative to self-reported preconception weight and adjusted for preconception weight reported at study entry rather than baseline weight. Demographic subgroup (i.e., age, ethnicity, parity, study entry weight, weeks gestation, income, site) main and interaction effects (with group × time) on postpartum weight over time from baseline were examined using the same likelihood-based, linear mixed effects models. Women who reported current pregnancy at 12 (*N* = 15), 18 (*N* = 5), 24 (*N* = 5) or 36 (*N* = 8) months postpartum were excluded from analyses of maternal weight (if provided) at those time points. Sensitivity analyses that excluded women who were pregnant at any time point yielded similar findings. The number of pregnancies did not significantly vary by randomized group.

For child outcomes, similar likelihood-based, linear mixed effects models were used for assessing the effects of treatment group on child BMIz and skinfolds, simultaneously adjusting for the same pre-specified potential effect modifiers as used in analysis of maternal weight. Multiple logistic regression analysis was used to examine the effect of treatment group on proportions of children at risk for obesity (>1 z score) [13] at 36 months with the same covariates. Demographic subgroup (i.e., maternal age, ethnicity, parity, study entry weight, weeks gestation, income, site) main and interaction effects (with group × time) on child BMIz over time were examined using the same likelihood-based, linear mixed effects models. Exploratory mixed model analyses examined relationships between gestational weight gain in kg during pregnancy and subsequent maternal weight and child anthropometrics over the postpartum time period. SPSS (25.0.0) was used for all analyses.

## Results

Figure 1 summarizes the participant flow in this study and number of participants in the mixed model analyses. The final 12-month visit in Healthy Beginnings/Comienzos Saludables was completed by 80.5% (*N* = 207/257) of participants. Out of the 257 participants in Healthy Beginnings, 243 (94.6%) were invited to participate in the follow-up study because they had a live birth, remained <36-months postpartum, and gave permission to be contacted for future studies. Of these, 173/243 (71.2%) met additional eligibility criteria and were enrolled in this follow-up study (113 enrolled and provided anthropometric data at 18 months, an additional 18 at 24 months, and an additional 34 at 36 months; enrollment time includes those providing chart-abstracted anthropometric data at that time point; 8 participants enrolled at 18 months, but did not provide any data). The primary reason for ineligibility in the follow-up study was loss of contact with the participant.

Of the 173 enrolled in the follow-up study, 165 (95%) completed at least one anthropometric assessment visit. Of those completing assessments, the 18 month anthropometric visit was completed by 105 (93%) of women enrolled and 113 (100%) of toddlers enrolled, the 24 month visit by 116 (89%) of women enrolled and 121 (92%) of toddlers enrolled, and the final 36-month visit was completed by 156 (95%) of women and 157 (95%) of toddlers. Participant characteristics in the follow-up study were nearly identical to those described previously for the full cohort [7] and were well balanced by randomized group (Table 1). As shown, 41.6% of the women were Hispanic/Latina, and 39.7% were with overweight and 60.3% with obesity.

**Table 1:** Characteristics of women and infants in Healthy Beginnings/Comienzos Saludables Follow-Up Study who enrolled at 18, 24, and 36 months.

	Standard care *N* = 85	Intervention *N* = 80
Maternal age (yr)	30.3 (5.5)	30.7 (5.3)
Hispanic	42.4%	46.3%
Married/living with significant other	89.4%	86.3%
Annual household income $		
≤$49,000	52.9%	56.3%
>$49,000	47.1%	43.8%
Some college or higher education	72.9%	78.8%
Employment		
Employed full time (at least 35 h.wk)	54.1%	57.5%
Employed part time (<35 h.wk)	20.0%	17.5%
Unemployed	25.9%	25.0%
Primiparous^b^	28.2%	30.8%
Gestational age at randomization (wk)	13.4 (1.9)	13.7 (1.6)
Weight at screening (kg)	87.8 (18.1)	84.9 (16.2)
BMI at screening (kg/m^2^)	33.1 (5.5)	32.8 (5.7)
BMI category at the screening		
Overweight	36.1%	44.3%
Obese	63.9%	55.7%
Prepregnancy self-reported weight (kg)	85.9 (18.4)	83.4 (16.4)
Prepregnancy BMI category (kg/m^2^)^a^		
Overweight	36.1%	44.3%
Obese	63.9%	55.7%
Infant characteristics		
Sex, male	56.5%	46.3%
Sex, female	43.5%	53.8%
Ethnicity, Hispanic	46.4%	46.2%

Data presented as percent or mean (standard deviation).

There were no statistically significant group differences. Enrollment in the follow-up study occurred at 18, 24, and 36 months.

^a^Note that these data are based on self-reported pre-pregnancy weight available in 83 standard care and 80 intervention participants. All women were with overweight or obesity based on the first prenatal, study entry measurement.

^b^Parity available in only 78 of the 80 intervention participants.

### Maternal outcomes

**Fig. 2:**
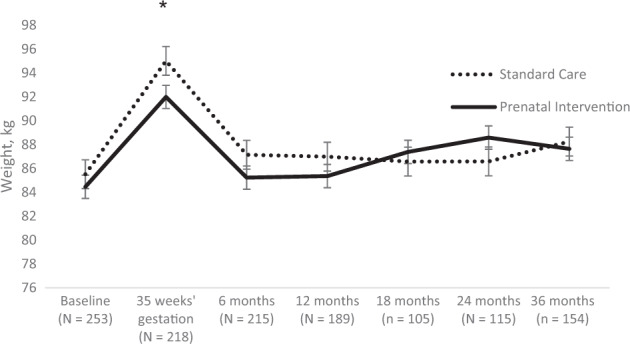
Maternal weight change in kilograms from early in pregnancy through 36-months postpartum. Data were adjusted for baseline covariates (age, weeks gestation at entry, income, ethnicity, parity, weight, and site). Participants missing baseline covariate data (*N* = 3) or who were pregnant at the time of any given assessment were not included in this analysis. A significant overall group × time effect was observed (*p* = 0.008); post hoc tests indicated difference at 35 weeks;* *B* = 2.1 [95% CI,3.4, 0.8]; *p* = 0.002.

In mixed effect models examining group effect on maternal weight from baseline through 36 months, a significant overall group × time effect was observed (*p* = 0.008). Subsequent post hoc tests revealed the intervention reduced weight gain during pregnancy (*B* = 2.1 [95% CI,3.4, 0.8]; *p* = 0.002), but did not significantly affect weight changes from baseline or from delivery through subsequent points during the postpartum period (Fig. 2). Similarly, at 36 months the intervention vs. usual care had no significant effect on the net (2.8 vs 3.0 kg) or percent (3.9 vs 3.4%) of weight retained relative to early pregnancy or on the proportion of participants who returned to their early pregnancy weight or below (33.3% vs. 39.5%; Table 2).

**Table 2:** Effect of Healthy Beginnings/Comienzos Saludables intervention on maternal postpartum weight retention at 36 months.

	Intervention (*n* = 72)	Standard care (*n* = 76)	Adjusted mean difference (95% CI)^a^	Adjusted odds ratio^a^
Postpartum weight retention at 36 months relative to baseline (i.e., early pregnancy) weight				
Net weight retention, kg (95% CI)	2.8 [0.8, 4.8]	3.0 [1.0, 4.9]	0.14 [−3.0, 2.7]	
Percent weight retention, % (95% CI)^b^	3.9 [1.7, 6.1]	3.4 [1.3, 5.6]	0.50 [−2.7, 3.7]	
At or below baseline weight, No. (%)	24 [33.3%]	30 [39.5%]		1.4 [0.7, 2.8]; *p* = 0.12
Postpartum weight retention at 36 months relative to preconception weight				
Net weight retention, kg (95% CI)	4.3 [2.2, 6.4]	4.3 [2.3, 6.3]	0.03 [−2.9, 2.9]	–
Percent weight retention, % (95% CI)^b^	5.9 [3.5, 8.3]	5.3 [2.9, 7.6]	0.57 [−2.8, 3.9]	–
At or below preconception weight, No. (%)	13 [18.1%]	27 [36.5%]	–	3.0 [1.3, 6.8]; p = 0.01

Data exclude women with current pregnancies at 36 months (*N* = 8). For analyses based on pre-conception weight, two participants in standard care did not report a preconception weight; thus sample size was 74 in that group.

^a^Parameter estimates with adjustments for baseline weeks gestation, age, income, ethnicity, parity, weight (either maternal weight at baseline or preconception), and site. Proportions are unadjusted.

^b^Percent weight retention was defined as postpartum weight retention divided by the starting weight used (either the maternal weight at baseline or the pre-pregnancy weight) and multiplied by 100.

Secondary analysis examined weight changes relative to self-reported preconception weight, and mixed effect models yielded similar results, showing effects during pregnancy but not beyond. At 36 months relative to preconception weight, the intervention had no effect on net or percent weight retention but statistically decreased the proportion of participants who returned to their preconception weight or below (18.1% vs 36.5%; *p* = 0.01; Table 2).

Group × time × demographic subgroup effects on postpartum weight from baseline were non-significant. A significant main effect for ethnicity was observed (*B* = −1.3 [−2.4, −0.3]; *p* = 0.02), indicating that Hispanic women retained more weight postpartum than non-Hispanic women (3.9 [2.1, 5.8,] vs. 2.2 [0.2, 4.1] kg, respectively). Significant main effects were also found for maternal baseline weight (B = −0.95 [−0.92, −0.98]; *p* = 0.0001), suggesting greater 36-month postpartum weight retention among women with lower weights at baseline. Greater postpartum weight retention was also observed among primiparous vs. multiparous women (4.6 [1.7, 7.4] vs 2.2 [0.8, 3.7] kg, respectively; *B* = 1.6 [0.5, 2.8]; *p* = 0.0001) and women with lower vs. higher incomes (4.2 [5.9, 2.4] vs. 1.4 [0.6, 3.4] kg, respectively; *B* = −1.2 [−2.3, −0.1]; *p* = 0.03).

### Infant outcomes

**Fig. 3:**
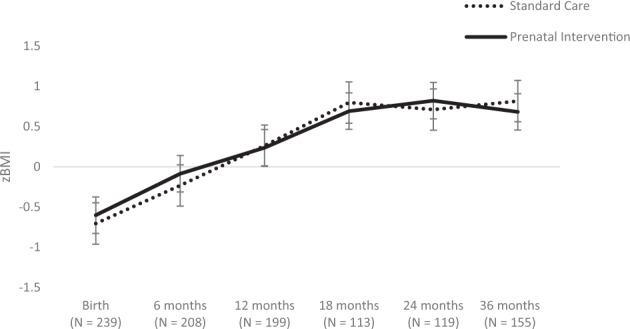
zBMI from birth through 36 months in children of intervention and usual care participants. Data were adjusted for baseline covariates (maternal age, weeks gestation at entry, income, ethnicity, parity, weight, and site). Participants missing baseline covariate data (*N* = 3) were not included in this analysis.

In mixed effect models, BMI z-scores (Fig. 3) and skinfold thicknesses increased (ps < 0.0001), but no significant group by time interactions were observed. From birth to 36 months, child BMIz increased by 1.4 [−1.7, 1.0] units in intervention and 1.6 [−1.2, 1.8] in usual care (Table 3). The proportion of children at risk for obesity at 36 months was similar in those randomized to intervention versus usual care (28/77 [36.4%] vs 30/80 [37.5%]; *p* = 0.77). Group × time × demographic subgroup effects were non-significant. A significant main effect for maternal age was observed, suggesting lower child BMIz with increasing maternal age (*B* = −0.3 [−0.05, −0.0007]; *p* = 0.007) but no other baseline covariates significantly predicted BMIz over time.

**Table 3:** Effect of Healthy Beginnings/Comienzos Saludables intervention on offspring anthropometrics through 36 months.

	Standard care *N* = 119*	Intervention *N* = 120*	Adjusted mean difference (95% CI)
Body mass index, z-score			
Birth	−0.7 (−0.9, −0.5)	−0.6 (−0.8, −0.4)	0.1 (−0.2, 0.4)
36 months of age	0.8 (0.6, 1.0)	0.7 (0.5, 0.9)	0.1 (−0.4, 0.2)
Change from birth to 36 months of age	1.6 (−1.3, 1.8)	1.4 (−1.7, 1.0)	0.2 (−0.6. 0.2)
Triceps skinfold, mm			
Birth	5.5 (5.1, 5.9)	5.2 (4.8, 5.6)	0.2 (−0.6, 0.1)
36 months of age	10.0 (9.5, 10.5)	9.3 (8.8, 9.7)	0.9 (−2.0, 0.0)
Change from birth to 36 months of age	4.6 (4.0, 5.3)	4.0 (3.4, 4.6)	0.6 (−1.5, 0.3)
Subscapular skinfold, mm			
Birth	5.3 (5.0, 5.5)	5.1 (4.9, 5.4)	0.1 (−0.5, 0.2)
36 months of age	6.2 (5.9, 6.5)	6.3 (5.9, 6.6)	0.1 (−0.7, 0.5)
Change from birth to 36 months of age	1.0 (0.6, 1.4)	1.0 (0.5, 1.5)	0.1 (−0.6, 0.7)
Thigh skinfold, mm			
Birth	7.0 (6.4, 7.6)	6.8 (6.3, 7.2)	0.2 (−1.4, 1.0)
36 months of age	13.4 (12.7, 14.2)	12.8 (12.2, 13.5)	0.6 (−1.9, 0.6)
Change from birth to 36 months of age	6.5 (5.7, 7.3)	6.0 (5.2,6.7)	0.3 (−1.4, 0.7)
Iliac crest skinfold, mm			
Birth	4.5 (4.1, 4.9)	4.5 (4.1, 4.9)	0.2 (−0.8, 1.1)
36 months of age	7.8 (7.2, 8.3)	7.7 (7.2, 8.2)	0.1 (−1.0, 0.9)
Change from birth to 36 months of age	3.3 (2.7, 3.9)	3.1 (2.4, 3.9)	0.1 (−1.0, 0.9)

Parameter estimates with adjustments for gestational age at randomization, age, ethnicity (Hispanic vs. non-Hispanic), parity, maternal baseline weight, income, and site.

*Longitudinal mixed model analysis included the full analytic sample (*N* = 239); sample sizes in standard care vs. intervention at each time point were 119 vs 120 at birth, 104 vs 104 at 6 months, 101 vs 98 at 12 months, 64 vs 49 at 18 months, 65 vs 54 at 24 months and 79 vs 76 at 36 months.

*SF* skinfold.

### Gestational weight gain and postpartum anthropometrics

Exploratory mixed model analyses, which included for group, group × time, and the usual covariates, indicated that gestational weight gain in kg during pregnancy was significantly related to greater subsequent increases in maternal postpartum weight over time (estimate = 0.4 [0.3, 0.6]; *p* = 0.0001]) and was weakly related to greater increases in child BMIz over time (*B* = 0.02 [−0.00, 0.04]; *p* = 0.06]). Similarly, exceeding NAS guidelines for gestational weight gain was related to greater maternal postpartum weight retention (estimate = 4.8 [2.7,6.9]; *p* = 0.0001] but not child BMIz (*p* = 0.12) over 36 months.

## Discussion

Although gestational weight gain is one of the strongest predictors of high postpartum weight retention, an intervention during pregnancy that effectively reduced gestational weight gain had no effect on maternal postpartum weight through 36-months postpartum. The prenatal gestational weight gain intervention also had no overall effect on child BMIz and skinfold thicknesses through 36 months.

To sustain improved maternal weight management initiated during pregnancy, continued intervention during the postpartum years may be needed [5]. In LIFE-Moms, four trials continued lifestyle interventions postpartum and three trials did not. In the trials with continued intervention, the frequency of intervention visits was reduced to monthly or less frequent, and the interventions continued to target healthy eating, physical activity, and weight control behaviors [10]. The trials with continued intervention during the postpartum period had the most enduring effects on reducing maternal postpartum weight through 12 months; effects were weaker in the three studies with interventions that were discontinued, including in the current trial [5]. Similarly, a Finnish trial found that a prenatal intervention that stopped at delivery (vs usual care) had no significant effects on maternal BMI after 7 years from delivery (27.3 vs 28.1 kg/m^2^, respectively) [14].

Although mothers face many barriers to continued weight management during the postpartum period, a variety of interventions, including internet-based lifestyle interventions, can effectively promote postpartum weight loss in diverse mothers [15–17]. In the current trial, postpartum weight gain appeared most pronounced after 12-months postpartum, suggesting a possibility that interventions could resume later in the postpartum year, after the initial dramatic changes of having a newborn have subsided. However, other studies that stopped intervention after pregnancy have reported weight gain beginning earlier in the postpartum year [18]. Ultimately, both intensifying interventions during pregnancy and continuing some form of treatment long-term might optimally reduce postpartum weight retention and related long-term comorbidities.

The effective prenatal gestational weight gain intervention had no overall effect on child BMIz or skinfold thicknesses through 36 months. The observed BMIz and skinfold values and changes over time were highly consistent with other studies of pediatric populations of women with obesity [18–22]. The observational literature and developmental origins of disease hypothesis suggest that reducing excess weight gain during pregnancy should lower offspring risk of obesity [3]. However, to date, prenatal lifestyle interventions have yielded mixed results on obesity risk during childhood [6]. Some positive effects were observed in a UK-based prenatal intervention targeting glycemic index, which also reduced gestational weight gain; the prenatal intervention reduced infant adiposity at 6 months [23] and had suggestive effects of lower odds of overweight/obesity at 3 years [22]. Also, a Finnish prenatal probiotic intervention designed to prevent allergic diseases in children reported a tendency for lower BMI in offspring at 4 years [9]. By contrast, LIFE-Moms [5] and other interventions [18] that effectively targeted and reduced excess gestational weight gain had no significant effects on child BMIz scores at 12 months. Prenatal intervention trials that targeted but had minimal to no effect on reducing gestational weight gain have also reported no effect on offspring BMIz at 6 months [24, 25] 18 months [26], and at 3 and 7 years [27–30].

Greater adherence to prenatal interventions has been related to greater long-term effects. One study found high vs. low adherence to a gestational weight gain intervention was related to reduced child BMI at 7 years (20.5 vs 22.5 kg/m^2^, respectively) [14]. To affect early metabolic imprinting and child obesity, more intensive maternal interventions might be needed [31] that are initiated earlier during pregnancy and that bolster adherence and promote greater reductions in gestational weight gain and fat mass. Also, interventions may need to be initiated preconceptually [32, 33] and/or continue to target maternal weight management during the postpartum years [34]. Continuing maternal intervention during the postpartum years could have a positive ripple effect on child BMIz [35]. Alternatively, combining maternal postpartum intervention with an effective pediatric preventive intervention to reduce obesity, such as a responsive feeding intervention [36], could enhance effects on child BMIz. It is clear that obesity risk in children is multifaceted and a number of factors early in life may also impact risk, including maternal and child feeding interactions [37], which will be examined in this cohort in a future study.

It is important to note that the intervention’s effects did not significantly vary across diverse demographic subgroups in analyses during pregnancy, at 12-months postpartum period [7, 8], and in this follow-up study. The intervention was designed to be linguistically and culturally relevant for diverse populations of women in California and Rhode Island. During development, significant efforts were made to ensure that the intervention was tailored for women with diverse ages, incomes, pregnancy histories, and cultural heritages. Overall, independent of group randomization, findings from the current study suggested greater postpartum weight retention among women with lower-income, Hispanic heritage, primiparity, and lower baseline body weight; these risk factors have been documented previously in observational studies [38]. Increasing age in the current study was related to lower child BMIz trajectories. These findings highlight a need for future research to develop and test effective interventions that reduce postpartum weight retention and overly rapid BMIz gain in the populations at greatest risk of obesity and related comorbidities.

Contrary to expectations, a secondary analysis of women who completed the 36-month visit suggested that this study’s prenatal intervention reduced the proportion of women who were at or below preconception weight at 36 months. Results relative to baseline weight were in the same direction, although not statistically significant. These results should be interpreted with caution, as they could be due to chance in the context of the high number of comparisons being made and/or reflect the reduced sample size present in this “completers” analysis at 36 months.

This study is one of the first to examine the long-term effects on maternal and child outcomes of an effective prenatal weight management intervention. The study included a diverse population and randomized design with highly trained assessors who were masked to randomization. This study also had some limitations. Only 66% of the initial sample was eligible and/or elected to enroll in the follow-up study; thus, sample sizes were smaller in the assessments beyond 12 months. Although practical, skinfold thicknesses are not an ideal representation of total body fat in children [19, 39]. Future studies should consider dual‐energy X‐ray absorptiometry (DXA) measures to quantify child risk of obesity.

In conclusion, excess gestational weight gain is a strong predictor of high postpartum weight retention and excess adiposity during toddlerhood. Although the intervention during pregnancy effectively reduced gestational weight gain, it resulted in similar maternal and toddler anthropometric outcomes at 36-month postpartum. Without continued intervention at 36 months, 64% of participants who received the prenatal intervention remained above their baseline weight and ~38% of toddlers were classified as having risk of overweight. Future research is needed to test the efficacy of combining more intensive prenatal lifestyle interventions with ongoing maternal/child care during postpartum years to reduce long-term excess weight gain and related comorbidities in diverse populations of women with overweight and obesity.
